# Winter Roost Preferences of Crows in Beijing City

**DOI:** 10.1002/ece3.70931

**Published:** 2025-01-28

**Authors:** Junyang Feng, Mengmeng Wang, Qianyi Zhang, Canwei Xia

**Affiliations:** ^1^ Ministry of Education Key Laboratory for Biodiversity and Ecological Engineering, College of Life Sciences Beijing Normal University Beijing China

**Keywords:** bird dropping, human–wildlife conflicts, noise, urban environment, urban management

## Abstract

During the winter season, an extensive population of crows (predominantly the carrion crow *Corvus corone* ) seek refuge in Beijing's urban landscapes for roosting, subsequently generating noise and droppings that adversely affect the quality of life of residents. This study elucidates the selection criteria employed by crows in Beijing's urban areas for roosting sites. Drawing upon historical records and the outcomes of our survey, we observed a remarkable consistency in the selection of roosting locations by crows over time, with the same locations being preferentially chosen across different years. We also conducted a quantitative analysis of the habitat characteristics associated with the crow roosting sites. We discovered that crows demonstrate a preference for roosting sites situated in proximity to human structures, particularly towering buildings, which are often adjacent to broad roads. By understanding the factors that influence the selection of roosting sites by crows, policy‐makers and urban planners can devise targeted interventions aimed at mitigating conflicts between humans and crows.

## Graphical abstract

The present study is centered on elucidating the selection criteria employed by crows in Beijing's urban areas for their night roosting sites, with the objective of providing empirical data to facilitate targeted management interventions aimed at mitigating the deleterious effects of crows. Drawing upon historical records and the outcomes of our comprehensive survey, we observed a remarkable consistency in the selection of night roosting locations by crows over time, with the same locations being preferentially chosen across different years.

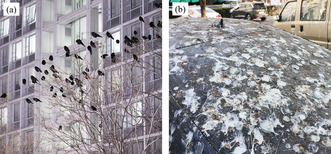

## 1 Introduction

Urbanization, fueled by rapid population growth and subsequent land use changes, poses significant challenges to terrestrial ecosystems worldwide (Šálek, Grill, and Riegert 2020). The transformation of natural habitats into urban areas drives land use changes (Pickett et al. 2001; Seto, Guneralp, and Hutyra 2012), leading to the decline and even local extinction of various species (Hahs et al. 2009; Piano et al. 2019; Silva, Sepúlveda, and Barbosa 2016). However, amidst these challenges, certain species, notably those with high adaptability, have found opportunities in urban and suburban settings (Neate‐Clegg et al. 2023). To thrive in urban environments, wild animals, including birds, often undergo behavioral changes. For example, birds in cities tend to engage in more group activities than their counterparts in natural habitats do (Gorenzel and Salmon 1995; Jaggard et al. 2015; Møller et al. 2019). One such behavior is communal roosting, which offers benefits such as thermoregulation (Beauchamp 1999; Du Plessis and Williams 1994; Putaala, Hohtola, and Hissa 1995), predation avoidance (Elgar 1989; Zuckerberg, McCabe, and Gilbert 2022), and improved foraging efficiency (Ward and Zahavi 1973; Evans 1982; Weatherhead 1983). While these changes allow birds to adapt, they may introduce challenges for urban residents.

The close proximity of large bird flocks to humans can cause problems, including noise disturbances (Peh and Sodhi 2002), disease transmission (Johan et al. 2022; Zhang et al. 2022), and collisions with vehicles (Duff 2013; Møller and Erritzøe 2017). Bird droppings can also negatively impact urban habitats and residents. The accumulation of bird droppings around habitats can pose health risks, as pathogens such as H5N8 avian influenza virus can survive in wild bird droppings for extended periods (Johan et al. 2022; Mitchell 2023; Zhang et al. 2022). In urban soil environments, bird droppings enrich mercury and increase the contents of heavy metals such as copper, zinc, cobalt, and nickel, which can migrate into plant systems (Fang et al. 2018; Yao et al. 2019). For historical buildings, cars, and power systems beneath bird roosts, droppings can decrease ornamental value, increase cleaning costs, and erode materials (Ali, Khattab, and Al‐Mukhtar 2015; Bernardi et al. 2009; Ramezanzadeh et al. 2009).

**FIGURE 1:**
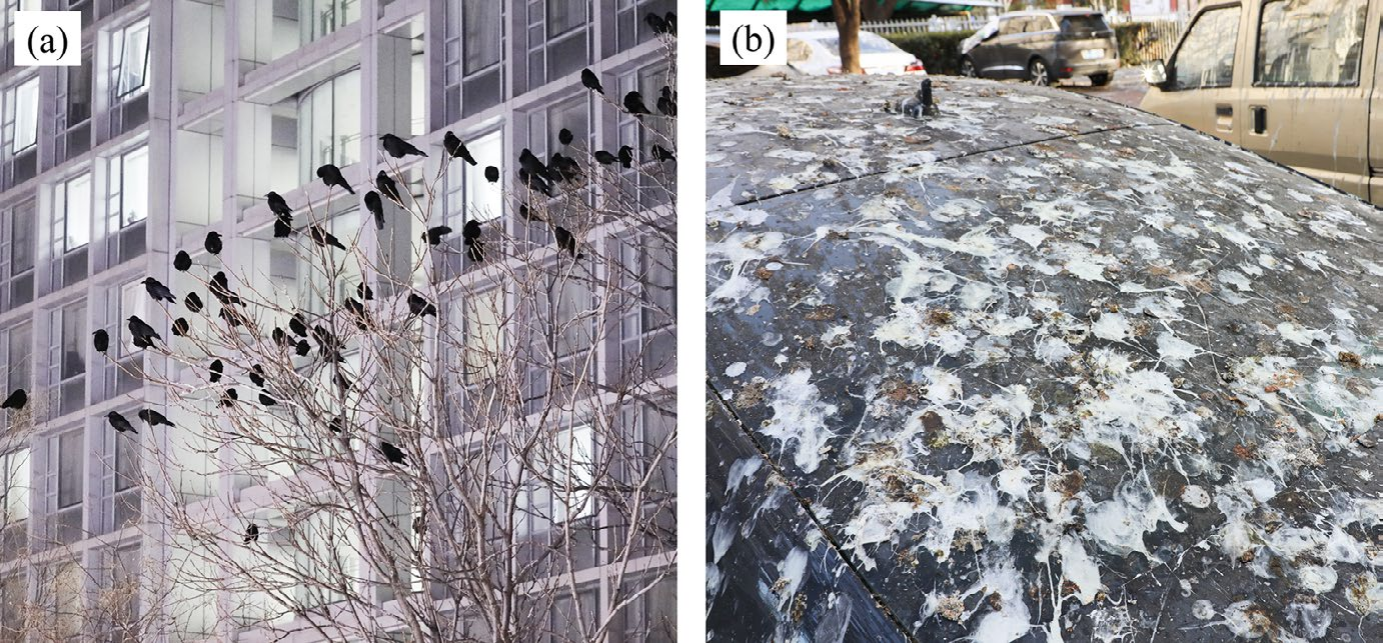
(a) Crows exhibit nocturnal roosting behavior within the urban area of Beijing; (b) A vehicle is extensively covered with crow droppings.

Crows (*Corvus* spp.) are “urban‐positive” birds that have successfully colonized urban environments and maintained large populations due to their substantial habitat and dietary plasticity (Gorenzel and Salmon 1995; Kövér et al. 2015; Peh and Sodhi 2002). Research by Benmazouz et al. (2021) revealed that the abundance and breeding success of 11 *Corvus* species increase with urbanization. This is evident in Beijing, a metropolis with approximately 22 million residents (Beijing Municipal Bureau of Statistics 2022), where significant numbers of crows roost in urban areas during winter (Figure 1), with up to 15,000 crows (predominantly the carrion crow *Corvus corone* ) counting as overwintering (Wang, Zhang, and Zhang 2016). Given the large and dense populations of both humans and crows, conflicts between people and birds can be pronounced. From 2014 to 2018, bird dropping flashover in the Beijing power grid accounted for 17 of 26 cases of bird damage (Yao 2019). Moreover, the old city of Beijing, dating to the Ming and Qing Dynasties (1368–1911), contains many ancient buildings worth protecting. Flocks of crows roosting in this area have increased the economic burden of maintaining these historic structures. Although crow droppings pose a hazard to urban residents, the affected locations are concentrated around the roosting sites of crows. These sites experience extended periods of crow activity, typically from after sunset until early the next morning, leading to more pronounced dropping accumulation (Wang, Zhang, and Zhang 2016). This localization allows for targeted prevention or mitigation of negative impacts.

This study examines the roosting preferences of crows within Beijing's urban landscapes, with a focus on two distinct spatial scales: a broader scale and a finer scale. The broader scale, encompassing areas ranging from hundreds to thousands of meters (hereinafter termed “location”), focuses on the overall distribution of crows in Beijing. In contrast, the finer scale (hereinafter termed “site”) examines the precise locations where crows seek refuge at night, aiming to identify areas where mitigation measures for human–bird conflicts may be needed. Based on our own observations in the field, we chose distances ranging from hundreds to thousands of meters as the broader scale because several consecutive roosting habitats can be contained within this distance, and at this scale there are clear and long intervals between flocks of roosting crows. Moreover, social learning among gregarious bird species facilitates the dissemination of behaviors, skills, and techniques across individuals and generations (Laland and Janik 2006; Aplin 2019). For example, New Caledonian crows ( *C. moneduloides* ) exhibit social learning in the creation and use of hook tools (Hunt and Gray 2003; Kenward et al. 2011), and many bird species, such as the black‐faced spoonbill ( *Platalea minor* , Li, Lu, et al. 2021) and the common whitethroat (*Curruca communis*, Tapia‐Harris and Cresswell 2022), demonstrate high spatial fidelity at nonbreeding sites. Based on this, we predict that crows' selection of roosting locations at the broader scale will remain stable over time, reflecting cultural inheritance, where the same roosting locations are chosen in different years. At the finer scale, we hypothesize that the impact of human activities on habitat characteristics is more pronounced. For example, the construction or demolition of parks can directly affect the habitats available for roosting birds. Communal roosting offers thermoregulatory benefits by minimizing energetic demands through huddling and reducing wind exposure, which is especially advantageous for species that spend the nonbreeding season in colder climates (Beauchamp 1999; Du Plessis and Williams 1994; Putaala, Hohtola, and Hissa 1995). Consequently, we predict that crows' selection of roosting habitats at the finer scale will take into account factors such as human disturbance and the need for warmth retention. This research examines the roost preferences of crows, which is conducive to targeted prevention and mitigation of human–bird conflicts, thereby fostering harmonious coexistence between humans and wildlife.

## 2 Materials and Methods

### 2.1 Study Site and Species

This research was conducted within the confines of Beijing (39°56′ N, 116°20′ E), a metropolis situated in the northwestern quadrant of the North China Plain. The city experiences a temperate continental monsoon climate characterized by oppressively hot and humid summers, which strongly contrast with extended and severely cold winters (He et al. 1993). Beijing has an annual mean temperature between 10°C and 12°C, with the lowest winter temperatures typically hovering at approximately −10°C and capable of decreasing to −20°C, and the annual precipitation in the urban area ranges from 400 to 800 mm (Beijing Municipal Bureau of Statistics 2022). This vast urban landscape encompasses a substantial building construction area of 129.626 million m^2^ and is inhabited by a population of 21.917 million residents (Beijing Municipal Bureau of Statistics 2022). The urban greening coverage rate in Beijing has reached 49.8%, with the primary species used for greening including evergreen vegetation such as pine trees (*Pinus* spp. and *Cedrus* spp.) and cypresses (*Platycladus* spp. and *Juniperus* spp.), as well as deciduous vegetation such as poplars (*Populus* spp.), ash trees (*Fraxinus* spp.), sophoras ( *Styphnolobium japonicum* ), ginkgos ( *Ginkgo biloba* ), and plane trees (*Platanus* spp.) (He et al. 1993). These vegetation types provide nocturnal habitats for avian species within the urban district of Beijing (Zhao et al. 2024).

The documented history of *Corvus* species inhabiting Beijing's urban landscapes dates to the Qing Dynasty (1644–1911), a period marked by the establishment of the “Suolun Pole” within the Forbidden City by the imperial court as a means of provisioning food for these birds. Scientific observations of the nocturnal roosting behavior of crows in Beijing's urban areas were recorded no later than 1956. Notably, Cheng, Li, and Zhou (1957) documented the diurnal foraging patterns of rook ( *C. frugilegus* ) in Beijing's suburbs, followed by their migration into the city's urban core for collective nocturnal roosting. Furthermore, a comprehensive survey reported by Wang, Zhang, and Zhang (2016) identified five distinct species of *Corvus* in the Beijing urban area in autumn and winter: carrion crow ( *C. corone* ), jungle crow ( *C. macrorhynchos* ), rook, Daurian jackdaw (*C. dauurica*), and collared crow (*C. pectoralis*). Research underscores the tendency of these five species to converge in sizable flocks for their nocturnal roosting, with the carrion crow emerging as a dominant component within these collective roosting groups (Wang, Zhang, and Zhang 2016). As stated in “Birds of Beijing” (Zhao and Zhu 2014), rooks and collared crows are relatively uncommon species in Beijing; jungle crows are abundant residents in the city, often observed alone or in small flocks; carrion crows and Daurian jackdaws are common winter visitors, with the former primarily roosting primarily in urban areas, whereas the latter prefer mountainous and hilly habitats in the suburbs. Thus, carrion crows are the primary species causing human–wildlife conflicts in urban Beijing.

### 2.2 Data Collection

In our study, we investigated the nocturnal roosting habitats of crows at two different scales. The broader scale focuses on the overall distribution of crows in Beijing; while the finer scale examines the precise locations where crows seek refuge at night, aiming to identify areas where mitigation measures for human–bird conflicts may be needed. At a larger scale, spanning hundreds to thousands of meters, we integrated historical records with field surveys. This approach enabled us to discern whether crows tend to roost in the same locations across different years. Conversely, at the finer scale, which focuses on the precise locations where crows take refuge at night, we conducted a rigorous quantification of environmental characteristics. The objective of this fine‐scale analysis was to gain insights into the criteria that crows employ in selecting their nocturnal roosting habitats.

**FIGURE 2:**
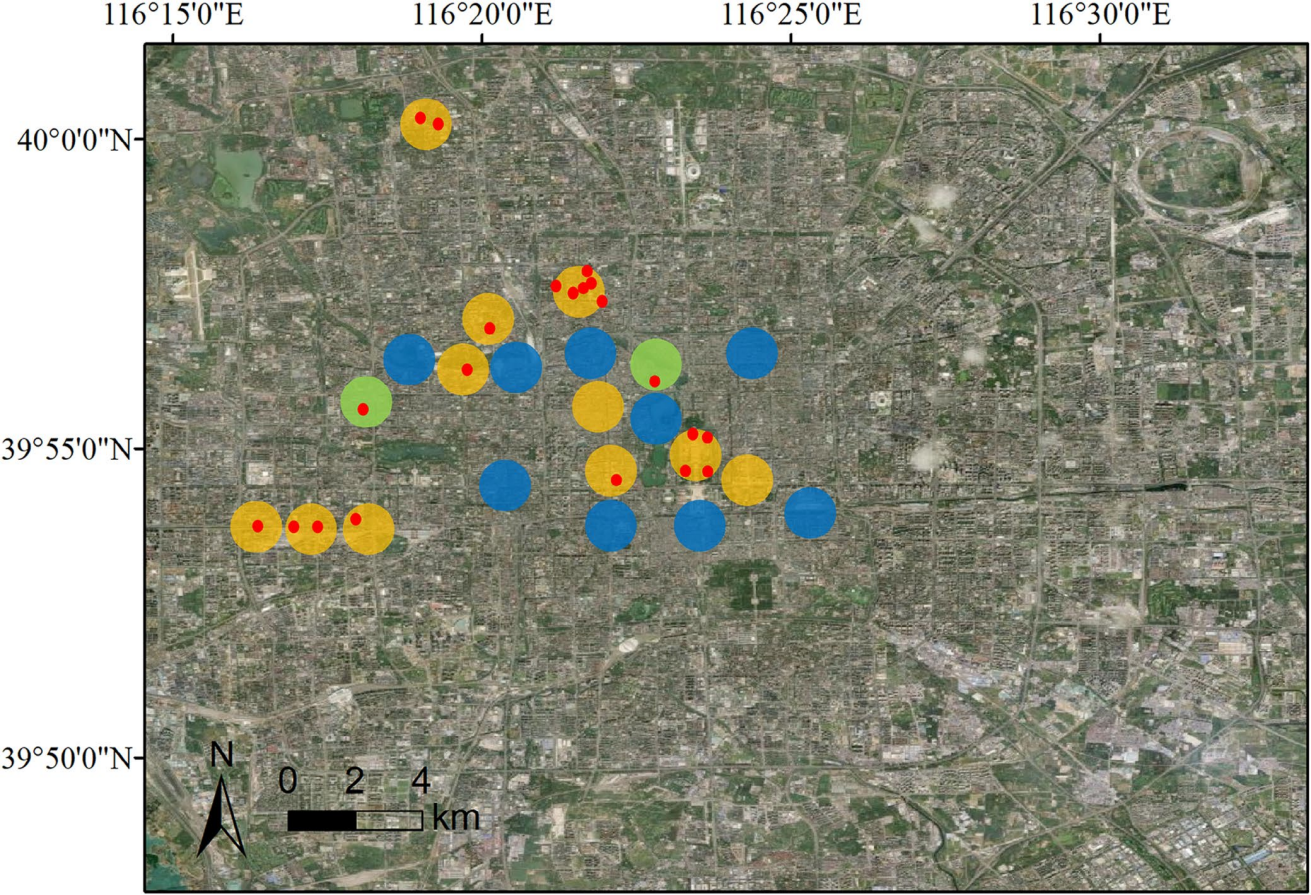
Roost site of crows in Beijing city. The large colored circles represent the locations we investigated, and the small red points represent the precise sites where crows take refuge at night. The orange circles represent the locations with concurrent confirmation of roosting crows by both historical records and our survey; the blue circles represent the locations with neither historical records nor observed crows during our survey; and the green circles represent the locations lacking historical records yet with observed crows during our survey.

Crows hold profound symbolic meanings in traditional Chinese culture; thus, their appearance in flocks is often recorded and reported by the public. During autumn 2018, we conducted a search on Baidu, the largest Chinese information search engine globally, employing the keywords “crow” (inclusive of all Chinese names, such as “WUYA 乌鸦” and “LAOGUA 老鸹”), in conjunction with “Beijing”. We reviewed the top 500 search results, focusing on those that (1) verifiably confirmed the existence of substantial flocks of crows, (2) depicted the birds engaged in nocturnal roosting behavior as opposed to mere overhead flight, and (3) provided sufficiently precise geographical locations. On the basis of these rigorous criteria, we successfully identified 11 distinct locations within Beijing's urban landscapes where crows were historically known to roost at night. Most of the recorded instances of crows roosting at these 11 locations can be traced to the 1970s and 1980s (Table S1). The earliest record dates to 1956, when Cheng, Li, and Zhou (1957) reported that crows roost at Beijing No. 3 Middle School. For each location with a historical record of roosting by crows, a control location was selected approximately 1 km peripherally from the original location. The control locations were chosen to have no historical record of roosting by crows and must not overlap. Four locations were situated in private residential areas and governmental departments where access was inconvenient, prompting us to appropriately extend the distance for selecting control locations. The minimum distance between the centers of adjacent locations was maintained at least 1 km apart (Figure 2).

We conducted comprehensive surveys at these 22 locations during the winter seasons of 2017–2018 and 2023–2024. The optimal timeframe for observing the nocturnal roosting sites of crows is during the evening hours, as it allows direct observation of the birds as they settle for the night. However, given the prevalence of private residential communities within the study area, accessibility during these hours is severely limited. Consequently, it was imperative to conduct the survey during daylight hours, when the crows had left their roosting sites for foraging in suburban areas. Therefore, we identified the crow roosting sites by observing their droppings on the ground. Flocks of roosting crows typically consist of hundreds to thousands of individuals (Cheng, Li, and Zhou 1957; Wang, Zhang, and Zhang 2016), a scale that is not reached by other common bird species in Beijing's urban areas. We used the presence of many droppings on the ground as the criterion for identifying crow roosting sites. When significant numbers of droppings were found, we took photos for recording and confirmed the location as a crow roosting site through conversations with nearby residents. Among the 22 locations mentioned above, if crows were observed to roost within a 500 m radius from the center of each location, it was recorded as a crow roosting location. Because we relied solely on droppings to determine the crow roosting locations and did not conduct molecular analysis, we were unable to record the specific species of crows or the exact number of roosting crows.

There may be multiple discrete roosting sites at each roosting location, reflecting the habitat requirements of crows in selecting roosting habitats. After field investigations of the roosting locations, 21 crow roosting sites were selected (Figure 2). To understand the habitat selection of crows for roosting sites, we conducted a survey of the habitats at these 21 crow roosting sites from December 30, 2017 to January 21, 2018. The following variables were recorded for each site: the presence or absence of tall buildings within 100 m of the roosting site in the east, west, south, and north directions; the height of the buildings; and the straight‐line distance from the buildings to the roosting site. At each roosting site, five trees were randomly selected where crows roost were measured for their circumference (measured 1.2 m from the ground), height, and crown cover area. For each crow roosting site, a control site without crow droppings was selected 200 m away along the road, and the same information was recorded. Owing to the absence of crows roosting at night at the control site, we randomly selected five trees for measurement of circumference, height, and crown cover area at the control site. If there are no trees available for crows to perch and roost at night at the control sites, the circumference, height, and crown cover area are recorded as 0. The variables involving distance are measured by a rangefinder with a precision of the meter level. The circumference of a tree is measured via a tape measure with a precision of the centimeter level. The crown cover area is measured by its projection on the ground.

### 2.3 Statistical Analyses

To rigorously examine the temporal stability of roosting location selection, we categorized the 22 surveyed locations into four distinct groups: those with concurrent confirmation of roosting crows by both historical records and our survey, those with historical records indicating roosting but no observed crows during our survey, those lacking historical records yet with observed crows during our survey, and those with neither historical records nor observed crows. By using Fisher's exact test to statistically compare the counts across these four categories, we can conclude that crows exhibit strong fidelity in their choice of roosting sites upon rejecting the null hypothesis (i.e., there is no association between historical records and our survey).

Furthermore, in our investigation of crow habitat preferences for roosting, we embarked on methodical preprocessing of the recorded variables. We derived metrics pertaining to the presence of tall buildings within a 100‐m radius in all cardinal directions (ranging from 0, there are no buildings in any direction within a 100‐m radius, to four, there are buildings in all directions within a 100‐m radius), the average and minimum distances from the roosting site to buildings (assigning a distance of 100 m in cases where no buildings are present within a 100‐m radius), the average and maximum heights of these buildings (likewise, assigning a height of 0 m where no buildings are present), and the average circumference, height, and canopy cover of the roosting trees. Additionally, we included the number of lanes on the nearest road to the roosting site, as recorded during our survey, resulting in a total of nine variables for subsequent analysis (Table S2). To mitigate the risk of Type I error inflation that could arise from individually comparing these nine variables, we employed principal component analysis, with rotation, to extract the most salient components. We subsequently utilized paired‐samples *t*‐tests to compare the differences in these principal components, with eigenvalues greater than 1, between the crow roosting sites and control sites.

All analyses were performed via R 4.4.1 (R Core Team 2024). The data are presented as the means ± standard errors, and *p* < 0.05 were considered statistically significant. When the statistical analysis involved requires the data to conform to normal distribution, the null hypothesis of normal distribution was not rejected (Kolmogorov–Smirnov test, *p* > 0.05).

## 3 Results

During the surveys conducted in the winters of 2017–2018 and 2023–2024, roosting of crow was observed at all 11 locations with historical records of crow roosting (Figure 2). In stark contrast, only two of the 11 control locations presented evidence of crow roosting in our survey. Statistical analysis revealed a robust association between historically documented crow roosting events and those identified in our current research (Fisher's exact test, *p* < 0.001). The investigations conducted across two winter seasons also demonstrated a remarkable consistency, where roosting locations identified for crows during the 2017–2018 winter survey were also found to be occupied by crows during the 2023–2024 winter survey, and vice versa. Despite the limitation of our investigations to two winter seasons, anecdotal evidence from residents proximate to the survey locations corroborated the continuous occupancy of these locations by crows for over a decade.

**FIGURE 3:**
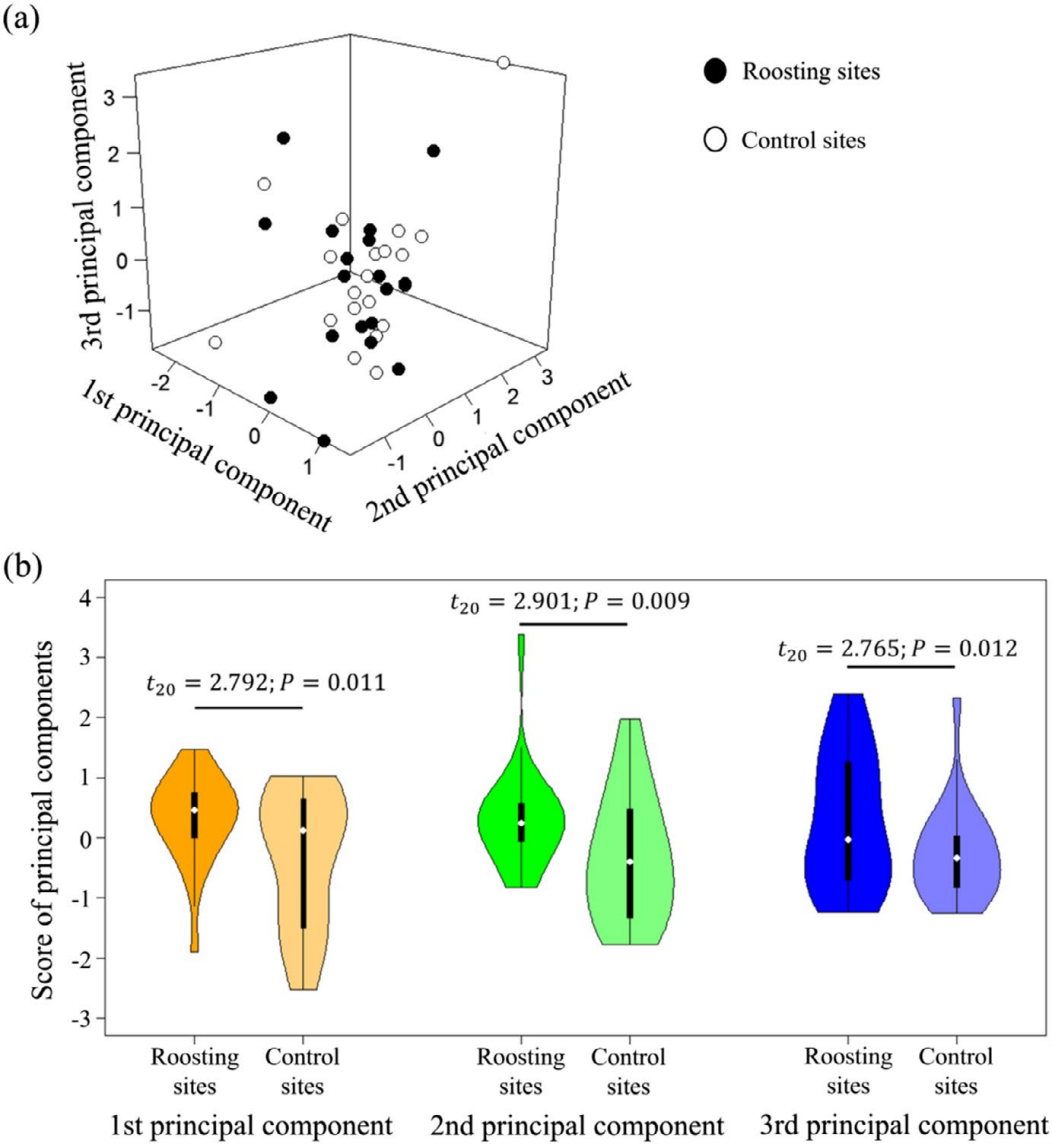
(a) Scoring of roosting sites and control sites (lacking crows) results in a degree of overlap across these three principal components (with eigenvalues > 1); (b) significant differences were found between these sites according to paired‐samples *t*‐tests. The biological significance of the principal components can be drawn from Table 1.

Principal component analysis yielded three components with eigenvalues exceeding 1, which collectively accounted for 82.5% of the total variability in the original dataset (Table 1). The first principal component was strongly correlated with the presence of tall buildings and the average and minimum distances from the roosting site to these structures. The second principal component was highly correlated with the average circumference, height, and canopy cover of the trees used for roosting. Last, the third principal component exhibited a significant association with the average and maximum heights of buildings in proximity to the roosting site, as well as the number of lanes on the nearest road. The scoring of roosting sites and control sites (lacking crows) revealed a degree of overlap across these three principal components (Figure 3a). However, significant differences were detected via paired‐samples *t*‐tests (Figure 3b), highlighting the preferential habitat selection patterns of crows at night.

**TABLE 1:** Principal component analysis for the nine variables concerning roosting habitats on three principal components (with eigenvalues > 1).

Variables	First principal component	Second principal component	Third principal component
Presence of tall buildings	**0.76**	0.05	0.30
Average distances to buildings	**−0.90**	−0.22	−0.13
Minimum distances to buildings	**−0.91**	−0.25	−0.20
Average heights of buildings	0.34	0.03	**0.88**
Maximum heights of buildings	0.40	0.00	**0.84**
Number of lanes on the nearest road	0.03	0.16	**0.73**
Mean circumference of roosting trees	0.05	**0.85**	0.40
Mean height of roosting trees	0.16	**0.94**	0.13
Mean canopy cover of the roosting trees	0.31	**0.82**	−0.25
Eigenvalue	2.62	2.41	2.41
Proportion of variance	0.29	0.27	0.27

*Note:* The absolute values of the correlation coefficients between the original variables and the principal components are displayed in bold when they are greater than 0.5.

## 4 Discussion

**FIGURE 4:**
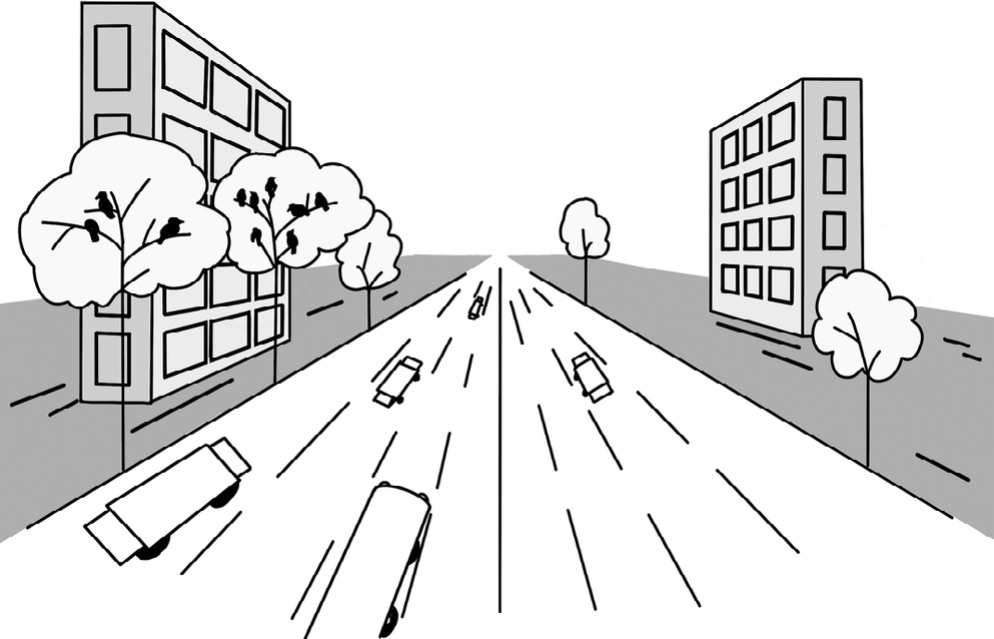
Schematic of the nocturnal roosting habitat of crows in the Beijing urban area. Crows choose to roost in tall trees near high‐rise buildings and broad driveways.

Crows constitute a prevalent avian species in Beijing's urban environments during the winter season. Our research revealed that these birds exhibit remarkable fidelity in selecting their roosting locations within the city's districts, with some locations being utilized consistently for more than half a century. On a microscale basis, crows demonstrate a preference for roosting sites situated in proximity to human structures, particularly towering buildings, which are often adjacent to broad roads (Figure 4). Moreover, the trees utilized for roosting purposes typically have considerable height and sturdiness. Furthermore, research conducted at both scales suggests that cultural inheritance and habitat characteristics may play crucial roles in the selection of roosting locations by crows. Consequently, understanding the roosting habitat preferences of crows in Beijing's urban areas during winter has potential for targeted prevention and mitigation of human–bird conflicts.

The phenomenon of bird fidelity to specific locations, including nesting sites, territories, and roosting sites, has been well documented in various studies. For example, the population of black‐faced spoonbill ( *Platalea minor* ) has high‐fidelity wintering habitats in China (Li, Lu, et al. 2021), and the population of common whitethroats (*Curruca communis*) has high spatial fidelity at nonbreeding grounds in Nigeria (Tapia‐Harris and Cresswell 2022). This behavior may stem from the need for optimal conditions that cater to the breeding, foraging, and roosting requirements of the birds. Given the substantial transformations that Beijing's urban areas have undergone over the past five decades, characterized by a doubling of the population and a more than tenfold increase in the built‐up area (Beijing Municipal Bureau of Statistics 2022), it is difficult to imagine that only these roosting locations have retained their attractiveness to crows over an extended period. Consequently, we postulate that the fidelity of crows to their roosting sites in Beijing's urban landscapes is likely propagated through cultural inheritance (Aplin 2019). For social bird species such as crows, young and inexperienced individuals often emulate the preferences of older, more experienced birds, thereby inheriting a preference for specific roosting locations (Brakes et al. 2021; Lewis 1996).

Furthermore, the selection of roosting sites near human structures and bustling main roads by crows may be influenced by their need for warmth during chilly winter nights in Beijing. Cities are known to exhibit a heat island effect, with heat sources including buildings and vehicle emissions (Botkin and Beveridge 1997; Guo et al. 2020; Li et al. 2020). The intensity of the heat island effect exhibits considerable heterogeneity within Beijing's urban areas (Sun and Shu 2007), where surface temperature variations during the same time in winter can range from 5°C to 9°C (Tuo 2017). The magnitude of warming attributed to the heat island effect is significantly correlated with the density of surrounding human structures and traffic congestion (Tao and Liu 2024). This additional warmth may be crucial for the survival of crows during the cold months in Beijing. Additionally, the presence of humans in these areas may offer crows a level of protection from predators, as wildlife often seeks refuge in human‐dominated landscapes to evade natural enemies (Møller, Díaz, and Liang 2016; Sakhvon and Kövér 2020). In the urban landscapes of Beijing, the risk of being exposed to predators can be lower (Sheng et al. 2024). Potential predators of roosting crows, such as the Siberian Weasel ( *Mustela sibirica* ), which comprises approximately one quarter of the winter diet with birds on the Tsushima Islands of Japan (Pyott et al. 2024), tend to favor secluded, dark areas devoid of human activity. Another predator that threatens bird survival is stray cats (Loss, Will, and Marra 2013; Li et al. 2021), with an estimated population of several hundred thousand in Beijing's urban areas (Jiang and Guo 2007). Although stray cats tend to be concentrated in urban districts, they are predominantly distributed in parks, residential communities, school campuses, and other relatively quiet locations during the night (Hou et al. 2009), with a tendency to avoid main trafficked roads in their activity ranges (Tatara and Doi 1994). By roosting in habitats adjacent to bustling main roads, crows can effectively alleviate the threat posed by these predators. Moreover, the preference for tall, sturdy trees as roosting sites serves a dual purpose for crows. It not only offers a physical barrier that minimizes disturbance from pedestrians but also may provide protection from ground‐based predators in urban areas (Peh and Sodhi 2002). Thus, the selection of such habitats serves as a reflection of thermal comfort and safety considerations, ensuring their survival and well‐being during the challenging winter season.

Beijing, as a megacity with a population exceeding 10 million, inevitably confronts the repercussions of night‐roosting crows on the daily lives of its urban dwellers. These consequences encompass disruptive noises emanating from crows during their arrival and departure from their habitats, which can significantly impair the sleep of residents, as well as the detrimental effects of crow droppings on buildings, vehicles, and crucial infrastructure such as power facilities (Ali, Khattab, and Al‐Mukhtar 2015; Bernardi et al. 2009; Peh and Sodhi 2002; Ramezanzadeh et al. 2009; Yao 2019). China implemented stringent wildlife protection measures, particularly after the COVID‐19 pandemic, which necessitated more rigorous scrutiny of wildlife management (Ministry of Ecology and Environment of the People's Republic of China 2023). Several commonly employed urban wildlife management strategies, such as capturing and relocating (Adams, Lindsey, and Ash 2016; Vandruff, Bolen, and San Julian 1996), can be implemented only during specific periods (e.g., avian influenza outbreaks) and in specific locations (e.g., airports), rendering their large‐scale application in urban Beijing almost infeasible. The present study reveals profound fidelity in the selection of roosting locations by crows within Beijing's urban landscapes, demonstrating a preference for habitats situated in close proximity to human‐constructed structures, adjacent to expansive roadways, and adorned with towering trees. This insight into the selection criteria of crows for their roosting sites offers valuable information for devising targeted strategies to mitigate the negative impacts of congregating night‐roosting crows on urban life. To address these issues, several preventive measures can be implemented. For example, the installation of protective covers on historic buildings and essential infrastructure, such as power facilities, located in the vicinity of crow roosting sites can effectively shield them from the corrosive effects of crow droppings. Furthermore, the incorporation of sound barriers in residential areas adjacent to these sites can significantly reduce the nuisance caused by the loud calls of crows, thereby enhancing the overall quality of life for urban residents. It is also necessary to investigate the perceptions and emotions of citizens regarding crows through questionnaires to determine a publicity plan (Špur, Pokorny, and Šorgo 2016; Kövér et al. 2022; Roshnath and Sinu 2024).

In conclusion, the primary objective of this research is to furnish foundational data that can inform strategies for mitigating the conflict between humans and birds, specifically by exploring the intricate selection processes of roosting habitats by crows within the urban fabric of Beijing. This approach contributes to a deeper understanding of the ecological behaviors of crows in urban environments and their implications for urban planning and management.

## Author Contributions

**Junyang Feng:** formal analysis (equal), investigation (equal), visualization (supporting), writing – original draft (lead), writing – review and editing (equal). **Mengmeng Wang:** conceptualization (equal), investigation (equal), writing – review and editing (supporting). **Qianyi Zhang:** investigation (equal), visualization (supporting), writing – review and editing (equal). **Canwei Xia:** conceptualization (equal), formal analysis (lead), funding acquisition (lead), project administration (lead), visualization (lead), writing – review and editing (lead).

## Conflicts of Interest

The authors declare no conflicts of interest.

## Supporting information


**Table S1.** Detailed information on the surveyed locations.

**Table S2.** Data on the environmental characteristics of the crows’ roosting sties and control sites.

## Data Availability

The dataset used in this study is included in Appendix.
